# Green Synthesis of Gold Nanoparticles with Curcumin or Açai in the Tissue Repair of Palatal Wounds

**DOI:** 10.3390/antiox12081574

**Published:** 2023-08-07

**Authors:** Anand Thirupathi, Morgana Francisco Machado Guzzatti, Maria Eduarda Anastácio Borges Corrêa, Ligia Milanez Venturini, Laura de Roch Casagrande, Igor Ramos Lima, Camila Da Costa, Ellen De Pieri, Lariani Tamires Witt Tietbohl, Paulo Emilio Feuser, Ricardo Andrez Machado-de-Ávila, Yaodong Gu, Paulo Cesar Lock Silveira

**Affiliations:** 1 Research Academy of Medicine Combining Sports, Ningbo No. 2 Hospital, Ningbo 315099, China; anand@nbu.edu.cn (A.T.); guyaodong@nbu.edu.cn (Y.G.); 2 Laboratory of Experimental Physiopathology, Program of Postgraduate in Science of Health, Universidade do Extremo-Sul Catarinense, Criciúma 88806-000, SC, Brazil; morganamachado@unesc.net (M.F.M.G.); meabcorrea@unesc.net (M.E.A.B.C.); ligia.milanez@posgrad.ufsc.br (L.M.V.); lauracasag@unesc.net (L.d.R.C.); igor.mamp@unesc.net (I.R.L.); camiladacosta49@unesc.net (C.D.C.); ellen.bn@unesc.net (E.D.P.); lariani.tietbohl@unesc.net (L.T.W.T.); paulo.feuser@posgrad.ufsc.br (P.E.F.); r_andrez@unesc.net (R.A.M.-d.-Á.)

**Keywords:** palatal wounds, inflammation, gold nanoparticles, curcumin, açai, green synthesis

## Abstract

This study aimed to evaluate and compare the effects of treatment with gold nanoparticles (GNPs) reduced with Curcumin (*Curcuma longa* L.) or Açai (*Euterpe oleracea*) to a standard commercial treatment of the pharmacological type (Omcilon^®^) and an electrophysical agent (photobiomodulation) in the palatal wounds of rats. As for the in vitro assay, a cell viability test was performed to assess the toxicity of the synthesized nanoparticles. In vivo assay: 60 Wistar rats were divided into five groups (n = 12): I. Palatal Wound (PW); II. PW + Photobiomodulation (PBM); III. PW + Omcilon^®^; IV. PW + GNPs-Cur (0.025 mg/mL); V. PW + GNPs-Açai (0.025 mg/mL). Animals were first anesthetized, and circular lesions in the palatine mucosa were induced using a 4 mm-diameter punch. The first treatment session started 24 h after the injury and occurred daily for 5 days. The animals were euthanized, and the palatal mucosa tissue was removed for histological, biochemical, and molecular analysis. GNPs-Açai were able to significantly reduce pro-inflammatory cytokines and increase anti-inflammatory ones, reduce oxidant markers, and reduce inflammatory infiltrate while increasing the collagen area and contraction rate of the wound, along with an improved visual qualification. The present study demonstrated that the proposed therapies of GNPs synthesized greenly, thus associating their effects with those of plants, favor the tissue repair process in palatal wounds.

## 1. Introduction

The integrity of the oral mucosa is important in preventing infections [1]. Oral ulcers are a type of lesion with a high prevalence rate in the oral mucosa. Oral disease hurts the patient’s well-being and quality of life [2].

Different treatments have been tested to avoid such complications and minimize the patient’s discomfort, but so far without absolute success. Advances in nanodentistry and innovations in oral health-related diagnostics, prevention, and therapeutic methods needed to maintain and achieve perfect oral health have been showcased. Recent advances in nanotechnology may bring about a paradigm shift in dentistry [3].

Among nanomaterials, metallic nanoparticles (NPs) have been considered for clinical application due to their low cost, high surface-to-volume ratio, high stability, and safety. Gold nanoparticles (GNPs) have many applications for therapeutic use in the biological detection of drugs and the delivery of proteins, genes, and drugs. These NPs have distinct properties and are being used in the areas of restorative dentistry, periodontology, dental implants, tissue engineering, and cancer diagnosis [4]. Gold-based nanoparticles exhibit unique electronic and optical properties, biostability, low cytotoxicity, and enhanced drug delivery capabilities, which open the possibility of their use in diverse fields. GNPs can penetrate the stratum corneum, and their drug delivery property makes them ideal as carriers for both large and small biomolecules [5].

The anti-inflammatory effects of GNPs are shown in the inhibition of the formation of pro-inflammatory cytokines in macrophages, a mechanism that is due to their ability to suppress the action of NF-κB in addition to their strong catalytic activity in the process of scavenging free radicals [6]. A recent study has observed that the topical application of GNPs in rats significantly accelerates the healing processes, performing wound closure four times faster, suggesting that gold nanoparticles are also capable of generating an increased proliferation of fibroblasts, which are important in the contraction of wounds [7].

Currently, the synthesis of nanoparticles from plant extracts is receiving increased attention due to its environmentally friendly behavior. The plant extract works as a reducing agent and a covering agent in the synthesis of nanoparticles [8]. Green synthesis, as it is called, is defined as the practice of using biologically friendly elements such as plants, bacteria, and fungi to synthesize nanoparticles. It is environmentally friendly and has no toxicity effects compared to chemical reduction methods. Biomolecules in the plant extract, such as amino acids, vitamins, enzymes, and proteins, work to reduce the concentration of metal ions [9].

Curcumin (*Curcuma longa* L.), a polyphenol extracted from the plant’s rhizome, is a pleiotropic molecule that can interact with several molecular targets related to inflammation. This molecule proved to be able to trigger early re-epithelialization, improve neovascularization, and increase the migration of various cells, including dermal myofibroblasts, fibroblasts, and macrophages, to the wound bed [10]. Açai (*Euterpe oleracea*) is a purple tropical fruit with many antioxidant nutrients and excellent anti-inflammatory and healing effects on the skin. Studies have shown that this fruit has effects in reversing mitochondrial dysfunction and anti-aging properties, in addition to also presenting an effect suppressor in the generation of reactive oxygen species and the activation of cyclooxygenase-2 (COX-2) and TNF-α in vitro [11]. 

Given what was presented above, the objective of this work was to evaluate the cytotoxicity and effects of treatment with gold nanoparticles reduced by the green synthesis method with Curcumin (*Curcuma longa* L.) or Açai (*Euterpe oleracea*) in the palatal wounds of Wistar rats compared to a commercial standard pharmacological treatment (Omcilon^®^) and an electrophysical agent (photobiomodulation).

## 2. Methods

All animal experiments were conducted in accordance with the National Institutes of Health’s (Bethesda, MD, USA) Guide for the Care and Use of Laboratory Animals and with the Ethics Committee of Universidade do Extremo Sul Catarinense—UNESC under protocol number 81/2022 in October 2021. Every animal experiment complies with the ARRIVE guidelines [12].

### 2.1. Green Synthesis of Gold Nanoparticles

GNPs were obtained by reducing different extracts. A stock solution of *Curcuma longa* L. (Sigma, São Paulo, Brazil) and/or *Euterpe oleracea* (Zona Cerealista, São Paulo, Brazil) extract was prepared in absolute ethanol. Stock solutions of chloroauric acid (HAuCl4) (4 mM) were prepared in Milli-Q water. Next, a pre-defined extract solution was added to Milli-Q water and kept under stirring and heating until reaching a temperature of 90 °C. Sodium hydroxide (NaOH) (0.1 M) was used to adjust the pH between 10 and 11. Next, a 1 mM solution of HAuCl4 was added dropwise under simultaneous stirring. The reaction was kept under stirring until reaching room temperature and the subsequent formation of GNPs (schematic representation in Supplementary Material).

The GNP solutions were characterized by ultraviolet-visible (UV-Vis) spectroscopy in a SpectraMax Plus model. Nanoparticles were measured in the visible region (390–700 nm). The size and morphology of nanoparticles were determined by transmission electron microscopy (TEM) using a JEM-1011 (100 kV). A drop of the nanoparticle solution was added to a copper grid (300 mesh) covered with a thin layer of carbon. Drying was performed at room temperature (24 h), and later images were obtained. Hydrodynamic size and surface charge at pH 7.4 (25 °C) were investigated by dynamic light scattering (DLS) and zeta potential measurements using a Zetasizer Nano ZS. The nanoparticle solutions were placed in a folded capillary cell. All analyses were performed in triplicate to obtain the mean and standard deviation (SD). The stability of the solutions was evaluated by UV-Vis and Zetasizer spectroscopy.

### 2.2. In Vitro Assay

#### 2.2.1. Cell Viability

For cell culture assays, an immortalized murine fibroblast cell line (NIH3T3) was used (Cell Bank of Rio de Janeiro, RJ, Brazil). Immortalized NIH3T3 cells were cultured in 25 cm^2^ plastic bottles with Dulbecco’s Modified Eagle (DMEM) medium supplemented with 1% penicillin and streptomycin antibiotics (10 U.L/mL) and 10% inactivated fetal bovine serum (complete DMEM).

For cell growth and adhesion, the cells were kept in a humidified incubator with an atmosphere of 5% CO_2_ at 37 °C. Medium changes were performed on alternate days, with the possibility of adaptations, until a sufficient cell confluence was obtained, around 80%, for the development of in vitro experiments. When the necessary confluence was obtained, the cells were trypsinized, the DMEM was removed from the bottle, and 4 mL of trypsin was added for 5 min, or until the cells were in suspension, detaching from the bottom of the bottle. Then, 4 mL of DMEM is added to neutralize the trypsin.

Then, the total number of cells was counted in the Neubauer chamber. After counting, these cells were diluted in a complete DMEM medium at the desired concentration. A total of 500 µL/well was added to a 24-well plate, with a final concentration of 1 × 10^5^ cells/well. Finally, they were incubated for 24 h in a humidified incubator with an atmosphere of 5% CO_2_ at 37 °C to ensure the adherence of the cells to the surface of the plate and then perform the cell assays.

After the cell adherence period, the complete DMEM medium was removed from all wells, and the following treatments were added to NIH3T3 cells: GNPs-Cur and GNPs-Açai at concentrations of 5%, 10%, 20%, and 30%. The solutions were diluted in DMEM. After the treatments for each group were added, the plate was incubated for 24 h in a humidified incubator with an atmosphere of 5% CO_2_ at 37 °C. The next day, the treatment was withdrawn, and then the reagent (3-(4,5-Dimethylthiazol-2-yl)-2,5-diphenyltetrazolium) (MTT) was added for the cell viability assay.

#### 2.2.2. Cell Viability Test—MTT

This method aims to evaluate cell viability by reducing MTT, which, in its oxidized form, is soluble in water and has a yellow color. 

After incubating the cells with the specific treatments for 24 h, a solution of 0.5 mg/mL of MTT diluted in PBS was prepared. The treatments were removed from the wells, and 100 µL/well of the MTT solution was added and incubated for 3 h (5% CO_2_ at 37 °C) to allow for the formation of formazan crystals. After the incubation period, the MTT solution was removed from all wells, and 100 µL/well of isopropyl alcohol was added to solubilize the formazan crystals. Finally, the plate was analyzed in a plate reader by absorbance at a wavelength of 570 nm. 

Cell viability is determined by comparing the absorbance results of the test groups (GNPs-Cur and GNPs-Açai) with the absorbances of the control group, that is, the group that has only viable cells. Therefore, the results are presented in percentages of cell viability, where the control group mimics 100% of live cells. Given this, calculations were performed to obtain the percentage of viable cells in each group, so that in this way the toxicity of the proposed molecule could be evaluated.

### 2.3. In Vivo Assay

#### 2.3.1. Animals

For this experiment, sixty Wistar rats (200–300 g, age 60 days) were needed. The rats were housed in particular cages with an ambient temperature between 20 and 22 ± /°C, a 12-hour light-dark cycle, a standard rodent diet, and water *ad libitum.*

The animals were randomly divided into the following 5 groups (n = 12):

- Palatal Wound (PW)—without local or systemic treatment;
- PW + Photobiomodulation (PBM)—standard treatment with Laser 660 nm 2 J;
- PW + Omcilon^®^—standard Omcilon^®^ treatment;
- PW + GNPs-Cur—treatment with gold nanoparticles reduced with curcumin (0.025 mg/mL);
- PW + GNPs-Açai—treatment with gold nanoparticles reduced with açai (0.025 mg/mL).

#### 2.3.2. Palatal Wound Model

The animals were anesthetized and maintained under anesthesia using ketamine 100 mg/kg and xylazine 0.5 mg/kg intraperitoneally. When the effect of the anesthetic was verified, each rat was stabilized, and the mouth was kept open using a retractor. The palatine mucosa close to the molar teeth was removed using a 4 mm-diameter stainless steel dermatological punch. The palatal mucosa involved in the circular incision was completely excised, and the periosteum remained exposed within this diameter. After bleeding control, the excised area was left exposed to simulate second-intention wound healing.

#### 2.3.3. Treatment

Twenty-four hours after injury induction, the animals in groups II, III, IV, and V were sedated with 4% isoflurane by inhalation and received their treatments. Group II underwent the first treatment session of photobiomodulation with an application of LBP AlGaInP with continuous emission (wavelength 660 nm; peak power 30 mW; beam size of 0.10 cm^2^, 2 J) from the company Laserpulse-Ibramed (São Paulo, Brazil). Irradiation was performed on the wound (five points around the wound), with the laser pen perpendicular to the palate at a distance of 0.5 cm per point, as described by Mendes et al. [7].

In the animals of group III, topical treatment (50 µL) with Omcilon^®^ was applied with a disposable micro-brush-type microapplicator on the palatal wounds. Groups IV and V received applications of GNPs-Cur or GNPs-Açai (50 µL hydrogel, dose of 0.025 mg/mL, and mean size of 20 nm), also with the disposable microbrush on the wounds. Treatments occurred daily for 5 days.

### 2.4. Macroscopic Analysis and Inflammatory Score

Macroscopic assessment of the wound relied on an overall visual score of the level of inflammation, with a protocol adapted from Marques Neto et al. [13], under blinding procedures by the applicators. 

The following parameters were included in the inflammation score:

- No change
- Irritation
- Shallow ulcer (1–2 mm) with clean edges
- Shallow ulcer (1–2 mm) with necrotic edges
- Shallow ulcer (3–4 mm) with clean edges
- Shallow ulcer (3–4 mm) with necrotic edges
- Deep ulcer (1–2 mm) with clean edges
- Deep ulcer (1–2 mm) with necrotic edges
- Deep ulcer (3–4 mm) with clean edges
- Deep ulcer (3–4 mm) with necrotic edges
- Necrotic tissue with increased margins

### 2.5. Euthanasia

Five days after injury induction and the daily application of treatments, the animals were anesthetized with 4% isoflurane and euthanized by decapitation. Next, the area of the injured palatal mucosa was excised with a remaining margin using a 5 mm-diameter stainless steel dermatological punch. The samples were then stored in individual flasks at 80 °C for further biochemical and histopathological analysis.

### 2.6. Wound Size Analysis

The photographic method is an accurate alternative to measuring the wound area. Images of the wounds were taken at a resolution of 3264 × 2448 pixels and analyzed using the ImageJ^®^ 1.51 software [14]. Images of the lesions were obtained on days 0 and 6 for visual verification of the evolution of the healing process and measurement of size (area in mm^2^) by calculating the variation in wound areas in this period in %. These measurements were made by the same researcher, with 3 measurements being performed for each wound and using the average value.

### 2.7. Histomorphometry 

Tissue samples from the soft palate (n = 5 animals/group) were soaked in a 10% paraformaldehyde (PFA) solution in 0.1 M phosphate buffer (pH 7.4) for 24 h. Subsequently embedded in paraffin after dehydration and bleaching and sectioned into 5 μm thick sections. To evaluate the quantification of the inflammatory infiltrate, hematoxylin and eosin (H&E) staining was used, and to assess and quantify collagenesis, staining with Gomori’s trichrome was used. The slides were read under an optical microscope (Eclipse 50i, Nikon, Melville, NY, USA) with a magnification of 600×. Images were recorded using a Nikon camera (Sight DS-5M-L1, Melville, NY, USA) and analyzed using NIH ImageJ 1.36b software (NIH, Bethesda, MD, USA). The count of the inflammatory infiltrate was performed using the Plugin “Cell Counter” of the software, considering the nuclear staining of inflammatory cells. To quantify collagenesis, the Plugin “Colour Deconvolution” of the software was used, which quantifies the percentage of blue color in the total area of the image.

### 2.8. Determination of Cytokine Content Using ELISA

The enzyme-linked immunoabsorbent assay (Duoset ELISA) capture method (R&D System, Inc., Minneapolis, MN, USA) was used to measure cytokines (TNF-α, IL1-β, IL4, and TGF-β). The samples were processed, and then the plate was sensitized for further incubation (at room temperature) with the antibody. 

### 2.9. Biochemical Analysis

- Intracellular determination of reactive oxygen species (ROS) and nitric oxide: The production of hydroperoxides was determined by the intracellular formation of 2′,7′-dichlorofluorescein (DCF) from the oxidation of 2′,7′-dichlorodihydrofluorescein diacetate (DCFHDA) by ROS according to the method previously described by Dong et al. [15] with some modifications. The production of nitric oxide (NO) was evaluated spectrophotometrically through the stable metabolite nitrite. The nitrite content was calculated based on a standard curve from 0 to 100 nM performed with the metabolite sodium nitrite (NaNO_2_). The results were calculated in μmol Nitrite/mg protein [16].
- Antioxidant defenses: Glutathione (GSH) levels were determined as described by Hissin and Hilf [17], with some adaptations. GSH was measured in the palatal mucosa homogenate after protein precipitation with 1 mL of 10% trichloroacetic acid. An 800 mM phosphate buffer, pH 7.4, and 500 μm DTNB were added to part of the sample. Absorbance was read at 412 nm after 10 min. A reduced glutathione standard curve was used to calculate the GSH levels in the samples.
- Protein Content: The protein content of the palatal mucosa homogenate was assayed using bovine serum albumin as a standard, according to Lowry et al. [18]. Phosphomolybdic phosphotungstic reagent (Folin phenol) was added to bind to the protein. Absorbance was read at 750 nm.

### 2.10. Statistical Analysis

The macroscopic data collected were analyzed using the IBM Statistical Package for Social Sciences (SPSS) version 21.0 software (IBM, 2012). Quantitative variables were expressed through mean and standard deviation, as they presented a normal distribution, and minimum and maximum values [19].

Statistical tests were performed with a significance level of α = 0.05 and 95% confidence. The distribution of data regarding normality was assessed using the Shapiro-Wilk test [20].

The comparison of the mean of the quantitative variables between the categories of dichotomous qualitative variables was performed using the Mann-Whitney U test, as the variable did not present a normal distribution. In turn, in the comparison of the mean of the quantitative variables between the categories of polyatomic qualitative variables, as they do not present a normal distribution, the Kruskal-Wallis H test was used, followed by the post hoc Dunn test when statistical significance was observed [21]. The investigation of the existence of an association between the qualitative variables was carried out by applying the Likelihood Ratio test, followed by residual analysis when statistical significance was observed [22].

The rest of the biochemical and molecular data were expressed as mean and mean standard error and statistically analyzed by two-way analysis of variance (ANOVA), followed by the post hoc Tukey test. The significance level established for the statistical test is *p* < 0.05. SPSS (Statistical Package for the Social Sciences) version 17.0 was used as a statistical package.

## 3. Results

### 3.1. Characterization of GNPs

The morphology and size distribution of the particles were analyzed using TEM, showing a spherical morphology and a size distribution in the range of 10–30 nm. Particle size distribution (PSD) and surface charge were determined by DLS. PSD was similar to the data presented using TEM. The zeta potential of the different nanoparticles was >−25 mV (pH 7), giving good stability to the different nanoparticles. A UV-Vis Spectrophotometer Analysis was performed, confirming the formation of different GNPs with curcumin and açai. Nanoparticles with different extracts do not show a significant difference (ANOVA *p* < 0.5) in the maximum wavelength. The obtained wavelengths confirm the formation of metallic nanoparticles. In addition, the stability of the GNPs was evaluated by UV-Vis for six months. The UV-Vis analyses showed that different GNPs remained stable over the six-month period. 

**Figure 1:**
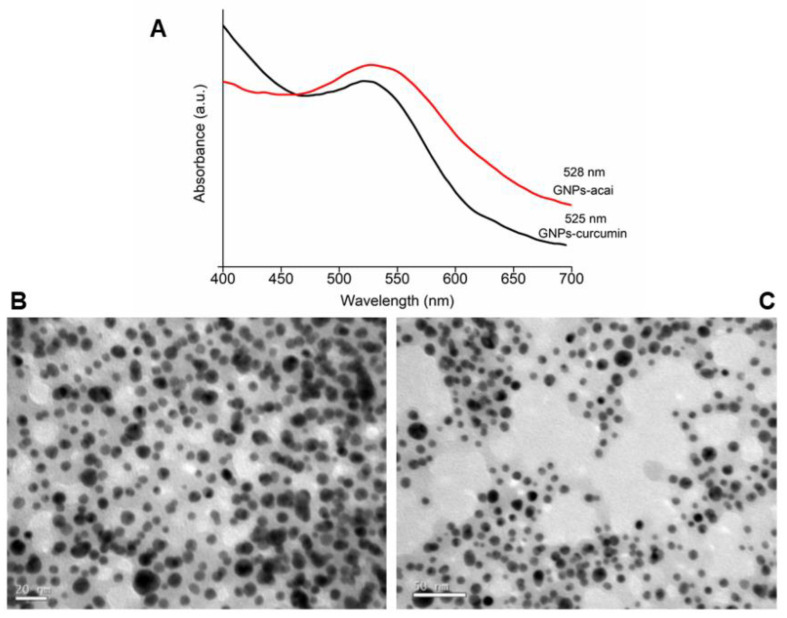
UV-Vis analysis (**A**) and TEM images of GNPs-Cur (**B**) and GNPs-Açai (**C**).

Figure 1A shows the maximum wavelength of both GNPs. Below are images of the nanoparticles obtained by TEM, in which Figure 1B is GNPs-Cur and Figure 1C is GNPs-Açai. Table 1 shows the results of the different nanoparticles’ mean size distribution and zeta potential.

### 3.2. Cell Viability Test

NIH3T3 fibroblast cells were exposed to four different concentrations of GNPs-Cur and GNPs-Açai. The tested concentrations were 5%, 10%, 20%, and 30%. The treatments occurred for an exposure period of 24 h. 

**Figure 2:**
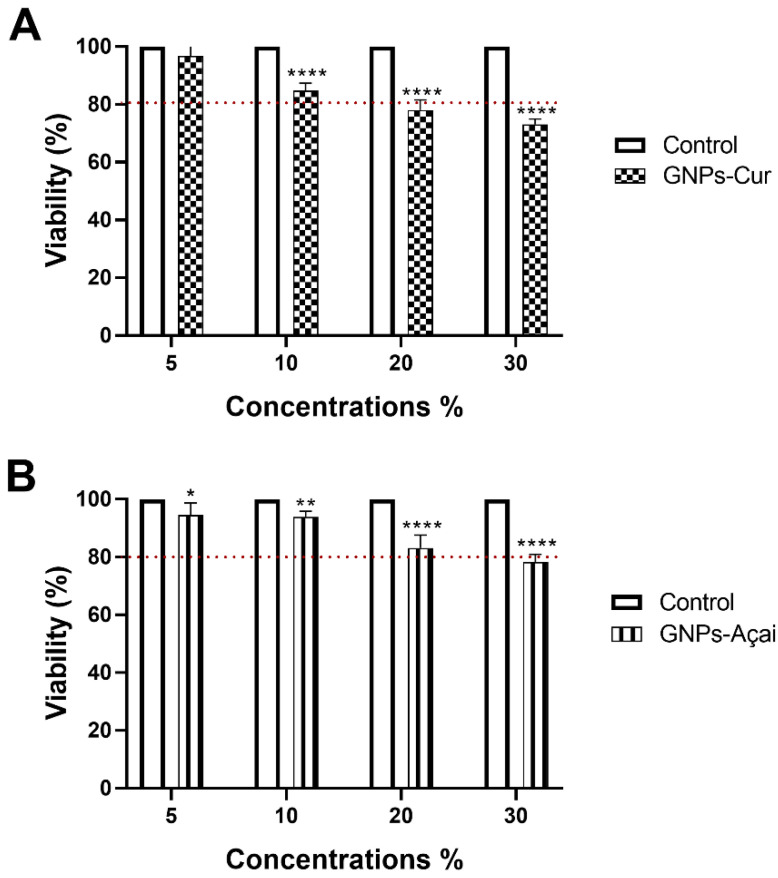
In vitro cytotoxicity of GNPs-Cur (**A**) and GNPs-Açai (**B**) at different concentrations evaluated in NIH3T3 cells within 24 h. Data are presented as mean + SEM, where: * *p* < 0.05 vs. Control; ** *p* < 0.01 vs. Control; **** *p* < 0.0001 vs. Control; (Two-way ANOVA followed by Tukey’s post hoc test).

Regarding GNPs-Cur, as can be seen in Figure 2A, at a concentration of 10%, there is a significant reduction when compared to the control; however, the viability is still above 80%. At concentrations of 20% and 30%, the viability is below 80%.

As for GNPs-Açai, in Figure 2B, concentrations of 5, 10, and 20% all presented significant reductions in relation to the control, although only at 30% the viability is below 80%. 

It is noteworthy that in both treatments, the concentration of 30% showed the lowest cell viability; that is, it was the most cytotoxic concentration for NIH3T3 cells. 

### 3.3. Macroscopic Analysis and Inflammatory Score

In Table 2, the value of * *p* = 0.027 in the comparison of treatments with GNPs reveals that this event did not occur by chance, indicating that the GNPs-Açai group obtained a better index of visual inflammatory condition than the other treatments.

### 3.4. Wound Contraction

**Figure 3:**
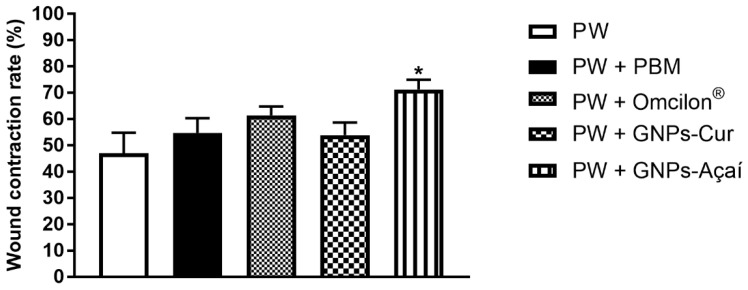
Effects of treatment with PBM, Omcilon^®^, GNPs-Cur, and GNPs-Açai on the wound contraction rate in %. Data are presented as mean + SEM, where: * *p* < 0.05 vs. Palatal Wound Group (PW); (One-way ANOVA followed by Tukey’s post hoc test).

Figure 3 represents the rate of wound contraction in %. There is a significant increase in wound contraction only in the PW + GNPs-Açai (*p* < 0.05) group in relation to the PW group.

### 3.5. Histological Analysis

**Figure 4:**
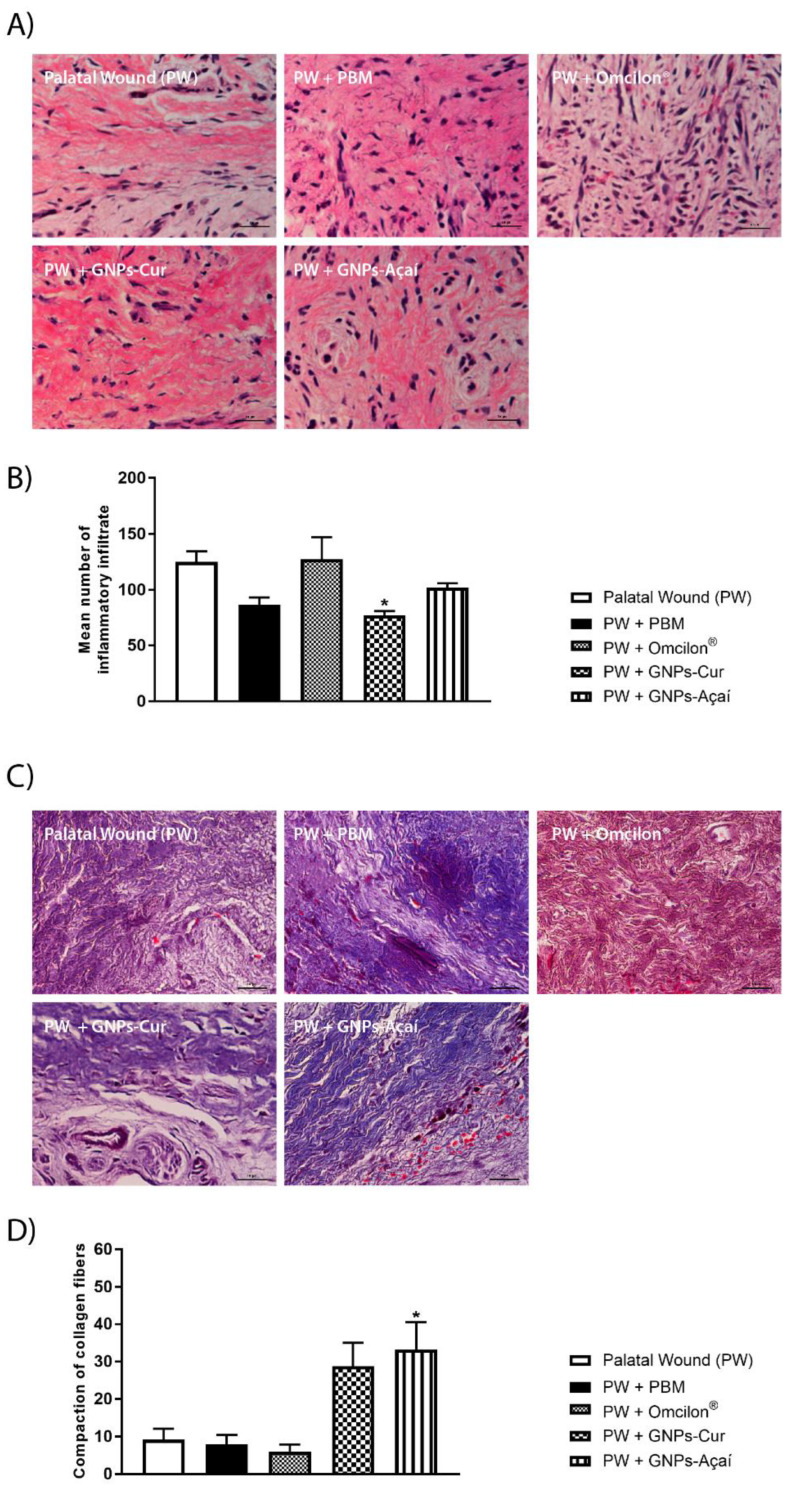
Effects of treatment with PBM, Omcilon^®^, GNPs-Cur, and GNPs-Açai on histological analysis. (**A**) Staining with H&E; (**B**) mean number of inflammatory infiltrate; (**C**) staining with Gomori’s trichrome; (**D**) compaction of collagen fibers. Scale 100 μm. Data are presented as mean + SEM, where: * *p* < 0.05 vs. Palatal Wound Group (PW); (One-way ANOVA followed by Tukey’s post hoc test).

In Figure 4A, representative images of histological sections of the palatine mucosa stained with H&E are observed. In Figure 4B, quantifications of the average number of inflammatory infiltrates were performed, with a significant reduction observed in the PW + GNPs-Cur group in relation to the PW group (*p* < 0.05).

Figure 4C shows representative images of histological sections of the palatine mucosa that were stained with Gomori’s trichrome to visualize collagen, and Figure 4D shows a significant increase in the area of collagen in the PW + GNPs-Açai group in relation to the PW group (*p* < 0.05).

### 3.6. Pro- and Anti-Inflammatory Cytokines

**Figure 5:**
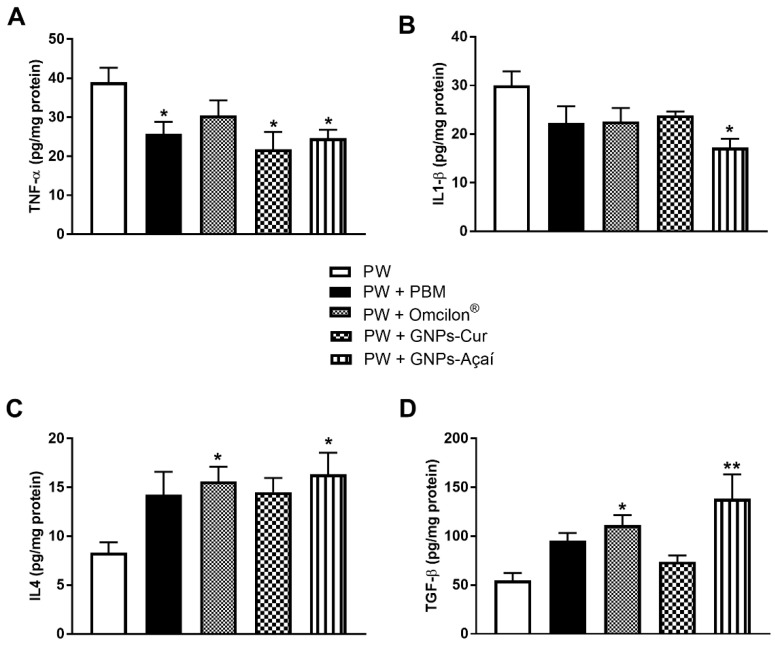
Effects of treatment with PBM, Omcilon^®^, GNPs-Cur, and GNPs-Açai on the levels of pro- and (TNF-α (**A**) and IL1-β (**B**)) anti-inflammatory cytokines (IL4 (**C**) and TGF-β (**D**)). Data are presented as mean + SEM, where: * *p* < 0.05 vs. Palatal Wound Group (PW); ** *p* < 0.01 vs. Palatal Wound Group (PW); (One-way ANOVA followed by Tukey’s post hoc test).

Figure 5 shows the protein levels of the cytokines TNF-α, IL-1β, IL4, and TGF-β. Representing the pro-inflammatory cytokines, when evaluating the levels of TNF-α, the PW + FBM, PW + GNPs-Cur, and PW + GNPs-Açai groups showed a significant decrease in relation to the PW group (*p* < 0.05). In Figure 5B, a significant reduction in IL-1β levels is observed in the PW + GNPs-Açai group when compared to the PW group (*p* < 0.05).

Regarding anti-inflammatory cytokines, Figure 5C shows the results of IL4, which showed a significant increase in the PW + Omcilon^®^ and PW + GNPs-Açai groups in relation to the PW group (*p* < 0.05), as well as what happened with TGF-β levels (Figure 5D), with a difference of *p* < 0.01 in the PW + GNPs-Açai group in relation to the PW group.

### 3.7. Levels of Oxidants and Antioxidants

**Figure 6:**
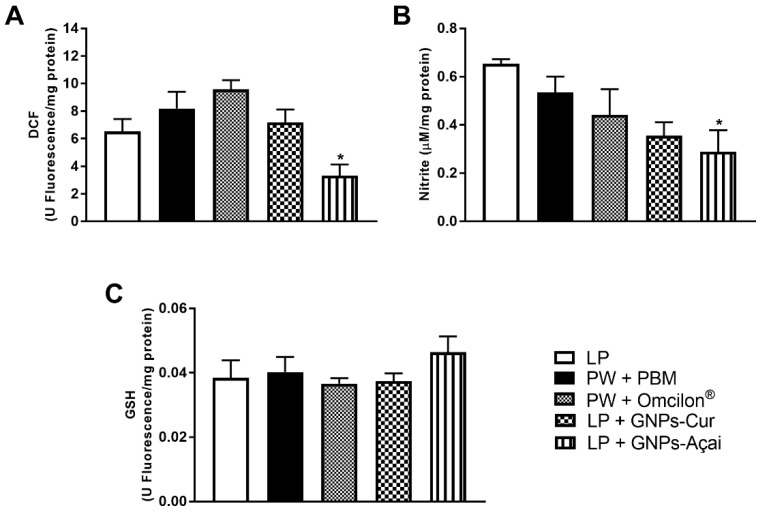
Effects of treatment with PBM, Omcilon^®^, GNPs-Cur, and GNPs-Açai on levels of oxidants (DCF (**A**) and Nitrite (**B**)) and antioxidants (GSH (**C**)). Data are presented as mean + SEM, where: * *p* < 0.05 vs. Palatal Wound Group (PW); (One-way ANOVA followed by Tukey’s post hoc test).

For the evaluation of oxidative parameters, the levels of DCF and Nitrite were analyzed, as was GSH as an antioxidant. In Figure 6A, DCF levels were reduced in the PW + GNPs-Açai group when compared to the PW group (*p* < 0.05), as was the case with nitrite levels (Figure 6B). GSH levels, on the other hand, showed no significant difference between groups.

## 4. Discussion

Due to the particularities and sensitivities that occur in the oral mucosa, causing some possible healing problems in the mouth, the literature is currently gaining attention to the development of biomaterials, such as nanoparticles, which may help trigger faster and more efficient healing. Because they have exceptional properties, a higher surface area/volume ratio, and different formats, NPs find applications in several fields of dentistry due to their mechanical, chemical, and optical properties. Gold is inert in nature, has low toxicity, and has also been used as a medicine. GNPs have many applications for therapeutic use due to their biostability, low cytotoxicity, and improved drug delivery capabilities, which enhance their use in various fields in addition to their potent anti-inflammatory and antioxidant effects [5].

The use of plant-based materials for the green synthesis of metallic nanoparticles has aroused much interest due to their lower toxicity, shorter processing times, and added benefit of being natural coating agents [23]. Curcumin, by limiting inflammation, allows damaged tissue to begin the later stages of healing sooner. The lack of proliferation and migration of fibroblasts will lead to impaired wound healing; curcumin upregulates fibroblast infiltration at wound sites [24]. Açai has antioxidant, anti-inflammatory, and anti-apoptotic effects in addition to promoting fibroblast proliferation; its supplementation can also protect against oxidative damage by reducing the formation of lipid peroxidation products, thus suggesting a potential protective effect promoted by the present antioxidants [25].

Thus, the present study evaluated the effects of treatment with GNPs reduced by the green synthesis method with curcumin and açai on the palatine wounds of Wistar rats. First, the inflammatory process of palatine wounds was evaluated using pro- and anti-inflammatory cytokines. Epithelial cells in inflamed regions express pro-inflammatory cytokines such as IL1-β, IL6, and TNF-α. This release leads to the activation of immune cells that accumulate in the inflamed sites and to the production and release of more cytokines [26]. Pro-inflammatory reactions after tissue injury are mainly triggered by IL1, which is associated with the innate immune system, and TNF-α, which is significantly involved in all inflammatory processes and whose release leads to vasodilation, accompanied by redness, swelling, and increased vascular permeability [27].

Both GNPs synthesized with curcumin and açai were able to significantly reduce the TNF-α marker in relation to the control, while only GNPs-Açai was able to reduce IL1-β. Regarding the anti-inflammatory cytokines, IL4, and the TGF-β growth factor, a significant increase was seen in relation to the control in the GNPs-Açai group, indicating that this therapy modulated and accelerated the inflammatory process, favoring the transition to the proliferative phase.

Controlling the overexpression of cytokines such as TNF-α may be useful in reducing pain and accelerating the healing of oral ulcers in rats, as it reduces acute inflammation [28]. In the case of GNPs, this anti-inflammatory action may be associated with their ability to suppress NF-κB [6], since it is known that inhibition of NF-κB activation leads to downregulation of the expression of COX-2 and inducible nitric oxide synthase (iNOS), suppressing the inflammatory response [29]. Another suggested mechanism is the direct binding of GNPs to IL1-β, inhibiting the binding of this cytokine to its receptor, as stated by Mendes et al. [7], who used gold nanoparticles in wound healing and also obtained a reduction in pro-inflammatory cytokines and an increase in anti-inflammatory cytokines, corroborating the present study.

In addition, in the recent study carried out by Ni et al. [30], it was demonstrated that GNPs can control the inflammatory response through the regulation of macrophage phenotypes (M1 and M2) and therefore generate a microenvironment with reduced levels of pro-inflammatory cytokines and an increased number of anti-inflammatory cytokines.

Wang et al. [31] synthesized curcumin solid lipid nanoparticles (Cur-SLNs) and increased their solubility compared to their native form and significantly reduced pro-inflammatory mediators by obstructing NF-κB activation, demonstrating the anti-inflammatory activity of this molecule. In vitro studies have also shown that Açai (*Euterpe oleracea*) extract downregulates NF-κB and its target genes, such as TNF-α, IL6, IL8, and IL1-β [32,33,34].

In the repair process, the inflammatory phase is accompanied by extensive phagocytosis, increased oxygen consumption, and mitochondrial dysfunction, which favor the formation of reactive species and enable a state of oxidative stress, which leads to a delay in the repair process. That is why, in this study, parameters of oxidative stress were evaluated. It was observed that GNPs-Açai were able to reduce both oxidant markers, DCF and nitrite, when compared to the control.

A benefit of açai is the ability to reduce the expression of the iNOS, contributing to the attenuation of the inflammatory response, in addition to the scavenging action (neutralizing free radicals) and the increase in the production and activity of antioxidant enzymes, such as superoxide dismutase (SOD), glutathione peroxidase (GPx), and catalase, which work in reducing ROS. Systemically, açai supplementation has been shown to influence the prevention of oxidative damage by a direct mechanism that reduces the formation of lipid peroxidation products, thus suggesting a potential protective effect promoted by the antioxidants present in açai (orientin, homoorientin, vitexin, luteolin, chrysoberyl, and quercetin [32,35].

The effective role of GNPs as antioxidant agents by inhibiting ROS formation, having a scavenger function (neutralizing free radicals), and potentiating the action of antioxidant enzymes has been demonstrated [7]. Li et al. [36] also demonstrated that GNPs increase levels of NRF2, which induces the signaling of antioxidant genes, in a study of endothelial cells. This increase was caused by the action of GNPs affecting the thiol bonds of KEAP1, thus changing its conformation and releasing NRF2 for subsequent transcription of cytoprotective genes, contributing to the antioxidant effect.

After the inflammatory process that takes place until the complete repair of a wound, it is expected that the repaired site will be restored to its normal anatomical condition as well as to its level of functionality. Collagen is the main element of the extracellular matrix and plays an important role in supporting mechanical loads by being a platform for re-epithelialization; therefore, its regeneration after injury is an essential process [37,38]. For this reason, in this study, the structure of the remodeled tissue was evaluated by histological analysis.

As in the studies carried out by BinShabaib et al. [39], Ning et al. [40], and Pona et al. [41], where there was a stimulus to collagen deposition, epithelialization, and wound contraction when evaluating the effects of *Euterpe oleracea* extracts on the healing process, the present study resulted in a significant increase in the area of collagen by GNPs-Açai in relation to the control. Raghuwanshi et al. [42] also demonstrated that GNPs were able to regulate collagen deposition, organize the extracellular matrix, and generate blood vessels, leading to rapid wound healing and closure.

Kang and Kim [11] show that there was a greater production and concentration of collagen in the oral mucosa of the group that received açai application, indicating that this molecule can prevent inflammation of the wound in the oral mucosa and promote re-epithelialization. In addition, it can help with mucosal regeneration and collagen synthesis at the time of remodeling.

As a result, in the results of the macroscopic analysis carried out in the present study to verify and record the visual perception of the inflammatory process, it was verified that the wounds treated with GNPs-Açai obtained an improved visual qualification, with an apparent reduction in the size of the wound and of the degree of inflammation and formation of necrotic tissue, corroborating with Weinberg et al. [43], who macroscopically evaluated the dimensional pattern related to the time taken to close an excisional palatine wound in rats, and obtained a macroscopic decrease in the dimensions of the wound in a temporal manner, which can be explained, as in the present study, by an acceleration of the inflammatory reaction and activity of myofibroblasts.

In this case, wound contraction is essential for reducing wound exposure to constant trauma and bacteria, which are characteristic of the oral environment. This contraction minimizes the open area by pulling neighboring tissue toward the center of the wound. Myofibroblasts, which arise by differentiation from fibroblasts, generate the contractile force by which the wound area contracts during wound healing [44]. However, a balance must be maintained, as although the contraction of the wound is beneficial in leading to its closure, excessive action can result in unwanted scars [45].

In the present study, the GNPs-Açai group was able to satisfactorily increase the wound contraction rate, which can be explained by the concomitant significant increase in the collagen area and the levels of the TGF-β growth factor, representing the potential of this cytokine to lead to re-epithelialization. Van Beurden et al. [46] point out that TGF-β has a biphasic role during the healing of palatal wounds since, at the beginning of healing, it stimulates myofibroblast differentiation and, after re-epithelialization, it stimulates myofibroblast apoptosis. Ratifying such findings, in the study by Kang and Kim [11], the aqueous extract of açai significantly reduced the surgical wound area performed on the backs of animals, showing a significantly potent wound healing effect in vivo based on macroscopic and histological observation. 

Correlating the histological, macroscopic, and wound contraction results, it can be seen that only the groups treated with açai obtained satisfactory results in all these parameters. It is thought that this substance may have some mechanism that allows it to present better visual results than at the biochemical level, since in the histological analysis when associated with GNPs, it significantly increased the area of collagen but was not able to significantly reduce the inflammatory infiltrate.

Importantly, the oral microbiota plays a dual role in both wound recovery and oral damage [47]. Lesions on the oral mucosa, despite suffering a relatively brief healing process compared to the skin, can still result in inflammatory responses that hinder the regeneration process. Current treatment models available on the market, such as interventions with “photobiomodulation” and topical use of synthetic corticosteroids, are not completely effective.

Lesions, however small they may be, are painful, making chewing, food intake, and communication very difficult. The present study pointed out that topical treatment with green synthesized nanoparticles is a potential resource for the recovery of palatal wounds and the treatment of oral mucosa lesions (such as ulcers), preventing exacerbated inflammatory responses or infections, and thus facilitating the healing process.

## 5. Conclusions

We believe that taken together, with the anti-inflammatory, antioxidant, and re-epithelializing effects of GNPs and açai, these associated substances could present statistically more significant results than the other treatments discussed, just as it is possible to state that the proposed treatment has better efficiency when compared to standard treatments used in clinical practice. However, future studies are needed to evaluate other mechanisms involved in palatal tissue repair and the use of these therapies in other experimental models of the oral mucosa.

## Figures and Tables

**Table 1:** Average size distribution and zeta potential of different nanoparticles.

Samples	SD (nm)	Zeta Potential (mV)	Maximum Wavelength
GNPs-Cur	39 ± 4	−22 ± 3	524 nm
GNPs-Açai	34 ± 2	−28 ± 3	526 nm

± Mean standard deviation of 3 determinations.

**Table 2:** Macroscopic analysis and inflammatory score.

GNPs	n	Mean ±	Minimum	Maximum	*p* Value
PW	9	3.22 ± 1.39	1	6	
PW + PBM	12	4.42 ± 3.11	1	10	
PW + Omcilon^®^	12	3.67 ± 2.06	2	9	
PW + GNPs-Cur	12	3.33 ± 1.96	1	7	
PW + GNPs-Açai	12	1.92 ± 0.79 *	1	4	0.027

## Data Availability

Data available on request from the authors.

## Supplementary Materials

The following supporting information can be downloaded at: https://www.mdpi.com/article/10.3390/antiox12081574/s1. Green synthesis of gold nanoparticles.
