# Supplementary figures and images for: Green Synthesis of Gold Nanoparticles with Curcumin or Açai in the Tissue Repair of Palatal Wounds
Source: Antioxidants (Basel). 2023 Aug 7;12(8):1574. doi: 10.3390/antiox12081574 (PMC10451912; doi:10.3390/antiox12081574)

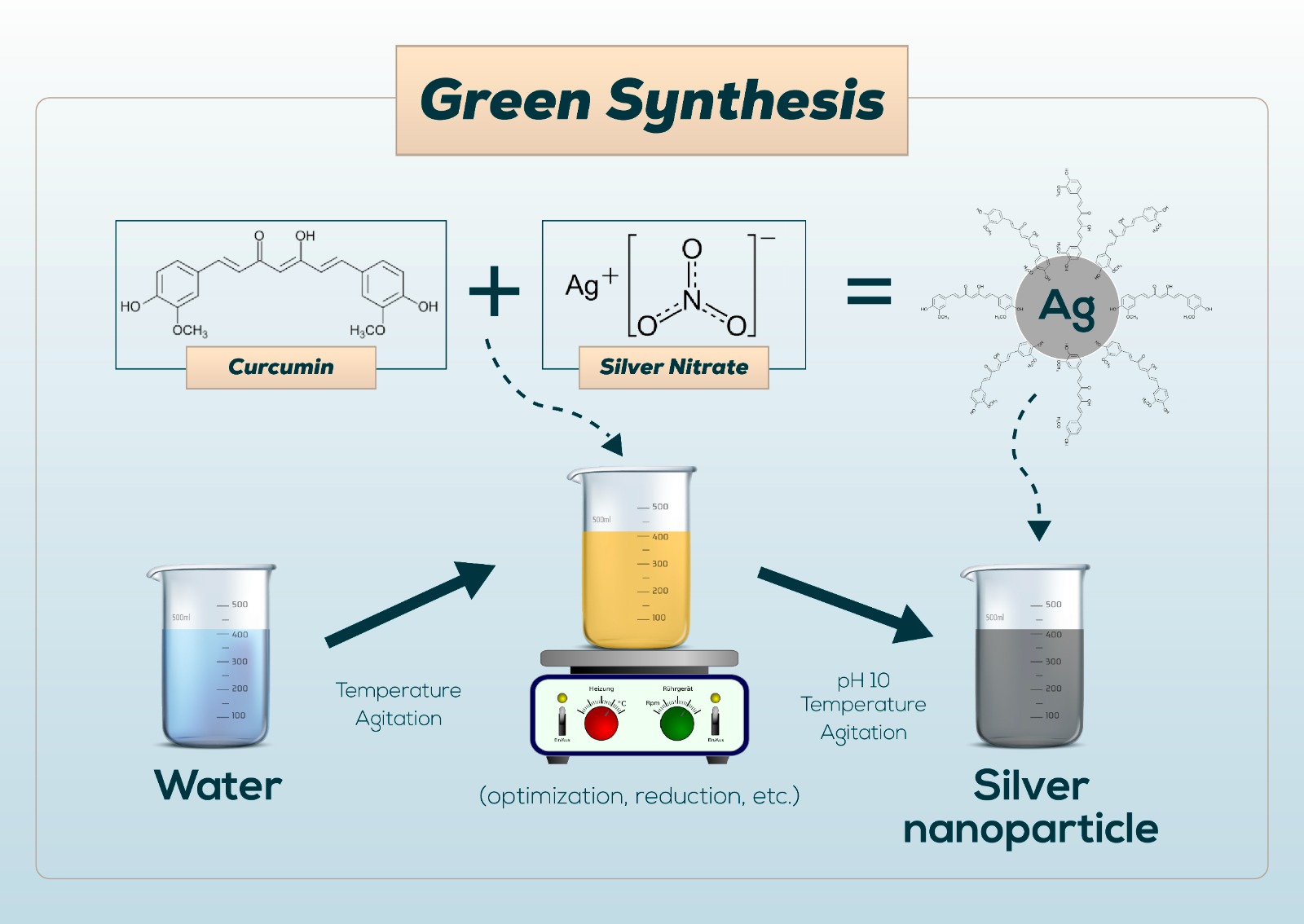

Supplement: Supplementary file 1 [file antioxidants-12-01574-s001.zip › antioxidants-2488825-supplementary.jpeg]
